# The Reliability and Validity of the Greek Version of the Child Abuse Report Intention Scale (CARIS) Questionnaire for Midwives, Along With Factors Associated With Their Intention to Report Child Abuse and Neglect

**DOI:** 10.7759/cureus.73500

**Published:** 2024-11-12

**Authors:** Eleni Theodoridou, Athanasios Sachlas, Alexandra Soldatou, Victoria Vivilaki, Angeliki Antonakou

**Affiliations:** 1 Department of Midwifery, School of Health Sciences, International Hellenic University, Thessaloniki, GRC; 2 Department of Computer Science and Biomedical Informatics, University of Thessaly, Volos, GRC; 3 2nd Department of Pediatrics, National and Kapodistrian University of Athens, School of Medicine, Athens, GRC; 4 Department of Midwifery, Faculty of Health and Caring Sciences, University of West Attica, Athens, GRC

**Keywords:** child maltreatment, identification, knowledge, midwifery, reporting

## Abstract

Objective: Child maltreatment is a serious public health issue with unquestionable short- and long-term consequences. The midwives' role in the prevention, identification, and reporting of child abuse and neglect (CAN) is crucial for children's well-being. The Child Abuse Report Intention Scale (CARIS) questionnaire was designed to measure factors influencing Taiwanese nurses to report child abuse and has been used in many studies worldwide. The purposes of this study were to test the reliability and validity of the Greek version of the CARIS questionnaire and to determine factors associated with the Greek midwives' intention to report suspected CAN.

Methods: A convenience sample of 70 registered midwives participated in the study from February to June 2023. After forward and backward translation, the participants completed the CARIS questionnaire twice, with a one-week interval, to determine test-retest reliability. Cronbach's alpha was used to assess the internal consistency of the questionnaire.

Results: Concerning the internal consistency, reliability, and repeatability of the Greek version of the CARIS questionnaire, our results were consistent, stable, and highly reproducible. For the CARIS, Cronbach's alpha ranged from 0.549 to 0.810 depending on the subscale, Pearson's statistic ranged from 0.649 to 0.797, while all the paired samples t-tests apart from one were nonsignificant. Concerning the subscale "Intended Practice Behaviors: Vignettes questions and Respond Options," Cronbach's alpha ranged from 0.527 to 0.890 depending on the subscale, Pearson's statistic ranged from 0.803 to 0.850, while all the paired samples t-tests apart from two were nonsignificant. Sixty-six participants (94.2%) had received no CAN training while in midwifery school. Only seven participants (10%) had ever reported suspected CAN, while 12 out of 70 (17%) did not report although they suspected CAN. Thirty-six midwives (51.4%) stated that they did not know how to report CAN cases, and 35 (50.7%) did not have the appropriate resources. Participants had overwhelmingly negative attitudes about child harsh discipline (34.34 of 36), low tolerance toward perpetrators (25.10 of 30), positive attitudes toward the responsibility of reporting suspected CAN (32.60 of 42), moderate knowledge of CAN (6.63 of 13), and increased engagement in reporting severe cases of CAN (32.37 of 40). Attitude, subjective norm, and perceived behavioral control were significantly related to the midwives' intention to report CAN.

Conclusions: The Greek version of the CARIS is a valid and reliable instrument that can be used to determine reasons influencing midwives' intention to report CAN. One of our key findings was the lack of adequate and appropriate training in dealing with CAN cases, both at the undergraduate and postgraduate levels, that needs to be addressed by health policy makers.

## Introduction

Child maltreatment is a public health issue with serious adverse health and social consequences concerning around 300 million children worldwide, according to the latest reports in 2023 [1,2]. In Greece, there is no established national recording system of child maltreatment cases yet. In 2021, the Institute of Child Health implemented an action called “Coordinated Response to Child Abuse and Neglect via Minimum Data Set” (CAN MDS). However, despite the pilot application of the CAN MDS system, it has not yet been implemented at a national level [3].

The most comprehensive study about the prevalence of children's exposure to violence in Greece is the Balkan Epidemiolocal Study of Child Abuse and Neglect. Greece was one of the nine participating Balkanian countries [4]. A key finding was that the ratio between self-reported cases of child abuse and cases of child abuse known to services during the same period from the same reference population and the same geographic area was less than 1% [3].

The iceberg phenomenon of underreporting child abuse is well documented [5,6]. While health professionals are in a unique position to safeguard children from maltreatment, there is a strong concern regarding their intention to report suspected child abuse and neglect (CAN) globally [7]. Identified reasons include inadequate knowledge of symptoms and vulnerability factors of CAN [8,9], ignorance of the legal framework and reporting process [10], uncertainty about corroborative evidence of CAN [11], negative experience with previous reports, including difference of opinion among involved health care professionals [12], and presence of a nonsupportive social environment [11]. In Greece, two additional factors have been identified: fear of child placement in an institution following a report to child protection authorities [13] and absence of a healthcare organizational CAN management protocol [14].

Midwives, as frontline health care professionals, are well placed to identify CAN and can play a key role in preventing CAN [15]. To our knowledge, potential factors that might influence midwives to report incidents of CAN have not been previously studied in Greece. The Child Abuse Report Intention Scale (CARIS) questionnaire was designed by Feng and Wu [9] and Feng and Levine [16] and has been widely used to identify factors associated with the intention to report CAN in other countries. This explorative pilot study aimed to translate and validate a Greek version of the CARIS instrument, assess its reliability for use by midwives, and identify the factors associated with midwives' intention to report suspected CAN cases.

## Materials and methods

Instrument

CARIS was designed by Feng and Wu [9] and Feng and Levine [16] to measure factors influencing Taiwanese nurses to report CAN. The questionnaire was based on the Theory of Planned Behavior [17] and consists of six sections: a) demographic and background information, b) attitudes toward CAN, c) knowledge of CAN and reporting laws, d) subjective norms regarding reporting suspected CAN, e) perceived behavioral control over reporting suspected CAN, and f) intended reporting behavior.

Demographic and background information include personal and professional characteristics, such as age, gender, marital status, years of professional experience, current job position, as well as any personal experience of CAN, such as being a victim or being related to a victim and any history of encountering and reporting suspected CAN.

Attitudes toward CAN are measured using three subscales that examine the following: a) attitudes regarding childbearing beliefs and discipline (six items), b) attitudes regarding punishment and culpability (five items), and c) attitudes regarding professional responsibilities (seven items). Participants indicate the degree to which they agree or disagree with statements on a 6-point Likert scale (from strongly disagree to strongly agree). Possible scores range from 6-36 for the first subscale, 5-30 for the second, and 7-42 for the third one. Negatively worded items receive reverse scoring (items a1, a2, a3, a4, a5, a6, b5, c3, c5, and c7). Higher scores indicate that participants have negative attitudes toward physical punishment, lower tolerance toward perpetrators, and positive attitudes toward professional responsibility to report suspected CAN.

Knowledge of CAN and reporting laws is measured using 13-item statements requiring a choice of one answer: a) I agree, b) I do not agree, and c) I do not know. Each correct answer counts as 1 point, and each incorrect or “I do not know” response counts as zero points. The range of the scale is 0-13, and a higher score indicates higher knowledge of child abuse and the reporting laws.

Subjective norms regarding reporting suspected CAN are measured by two statements, which examine the perceived social pressure to report or not an incident. Participants indicate the degree they agree or disagree with statements on a 5-point Likert scale (from strongly disagree to strongly agree) and scores range from 2-10. The higher the score, the higher the perceived pressure to report.

Perceived Behavioural Control over-reporting suspected CAN is measured using eight items, scored on a 5-point Likert scale ranging from “definitely no” (1 point) to “definitely yes” (5 points), and refers to the confidence to report suspected CAN. Negatively worded items are reverse scored (items 2, 4, 7, and 8), and the possible score ranges from 8 to 40. As the score increases, participants perceive a greater sense of control over the reporting process.

The Reported and Intended Behavior Scale is based on participants' responses to eight vignettes consisting of one severe and one less severe case of four different types of child maltreatment (neglect, physical abuse, psychological abuse, and sexual abuse). Participants rate their intention to report by using a 10-point Likert scale from "almost certainly would not report" (1 point) to "almost certainly would report" (10 points), and the total score is calculated for four types of abuse cases at two levels of severity. A higher total score reflects a greater intention of reporting.

CARIS showed evidence of construct validity and reliability and a final Cronbach's alpha for the subscales ranging from 0.62 to 0.91 [16]. Since its initial evaluation, CARIS has been translated and used in numerous studies worldwide [9-11,16,18-24].

Procedure

The scale's creator, Feng, granted permission to use the CARIS questionnaire by email. The translation proceeded according to World Health Organisation guidelines for the translation process and adaptation of instruments [25]. A group of five Greek experts, including three midwives, a pediatrician, and a lawyer with a special interest and expertise in child abuse, were asked to review the tool for content validity. Content validity indices were calculated based on experts' agreement. A few adjustments were needed to adapt to the Greek language, culture, legal framework, and education system. For instance, in Greece, midwifery is a different profession from nursing, so every "nurse" word was replaced by the word "midwife." Additionally, midwifery is not divided into specialties, so question 12, referring to nursing specialties, was removed from the professional information section. Question 5 about different religious doctrines was removed from the initial questionnaire since more than 95% of the Greek population is self-defined as Christians. Furthermore, given midwives are not mandatory reporters of suspected CAN, according to the Greek legislation, statements 1 and 12 from the “Knowledge about CAN and the reporting law” sector were scored as false. Finally, to increase the accuracy of responses in Greek, we changed the rating scale in the Perceived Behavioral Control section, which measures the perceived behavioral control, from "definitely no and definitely yes" to "strongly disagree and strongly agree." All the above adjustments were approved by the initial scale's creator.

Subsequently, ethical approval was requested and obtained from the International Hellenic University's Research Ethics Committee (approval number 20 dated February 02, 2023) prior to conducting the study, which is a part of the Ph.D. project of the principal investigator (E.T.).

All registered midwives, qualified and working in Greece and willing to participate, were considered eligible to take part in the study. All respondents were informed about the volunteer and confidential nature of participation in the study. No identifiable data were collected, and participants were free to withdraw at any time without consequences. Approvals were obtained from the scientific councils of two maternal hospitals in Athens. To calculate test-retest reliability, midwives were asked to fill in the questionnaire twice at a one-week interval.

According to the formula for calculating the sample size in pilot studies proposed by Viechtbauer et al. [26], a sample of at least 59 participants is required to identify problems with a prevalence of 5% (with 95% confidence). A convenience sample of 70 individuals was selected for this study, which was conducted in Athens, Greece, from February to June 2023.

Statistical analysis

Absolute and relative frequencies were calculated for qualitative variables, while central tendencies and dispersions were calculated for quantitative variables. To test the internal consistency of the questionnaire's subscales, Cronbach's alpha coefficient was calculated. Pearson's linear correlation coefficient was used to test repeatability, while the intraclass correlation coefficient (ICC) and t-test for dependent samples were used to assess test-retest reliability. Statistical analysis was performed using the IBM SPSS Statistics software program, version 29.0 (IBM Corp., Armonk, NY) for Windows, while the significance level for all analyses was set at 5%.

## Results

Participants' characteristics

The total sample consisted of 70 registered midwives (68 women, 97.1%) with an average age of 39.55 (±8.48) years. More than half (37) were married (52.9%), and 36 midwives had children (51.4%). The average interval from the participants' last degree was eight years (Table 1). The average professional experience was 14.86 (±8.82) years, with 40 midwives (57%) working in the private sector, 15 (21.5%) in the public sector, and 15 (21.5%) as freelancers. During the study period, 55 (78.6%) respondents were working as clinical midwives, seven (10%) as supervisor midwives, two (2.9%) as educators, and six (8.5%) in some other midwifery sector. Participants provided healthcare to 11.90 pregnant/postpartum women/mothers per day on average.

**Table 1:** Participants' characteristics

Characteristics	Values
Sex, n (%)	
Women	68 (97.1)
Men	2 (2.9)
Age, mean (± standard deviation)	39.55 (±8.48)
Marital status, n (%)	
Single	23 (32.9)
Married	37 (52.9)
In a permanent relationship	3 (4.3)
Separated	1 (1.4)
Divorced	6 (8.6)
Having children, n (%)	
No	34 (48.6)
Yes	36 (51.4)
Number of children, n (%)	
1	12 (34.3)
2	21 (60.0)
3	2 (5.7)
Academic status, n (%)	
Bachelor’s degree	43 (61.4)
Master’s degree	36 (37.1)
Doctorate of philosophy	1 (1.4)
Years since their last degree, median (± interquartile range)	8.0 (±14.0)

Regarding child maltreatment, 65 participants (92.9%) stated that they did not have any personal adverse experiences as children, but 23 midwives (32.9%) knew someone who was abused as a child. Seven out of 70 (10%) had reported suspected CAN cases in the past, while 12 participants (17%) had opted not to report despite suspicion. When asked about the reasons for not reporting, participants mentioned "feeling uncertain about the evidence" and "lack of trust in legal authority" as the main reasons. The great majority of the participants (66 of 70, 94.3%) had received no training about CAN during their studies, only two (2.9%) had received a three-hour relevant training, one person (1.4%) had received a four-hour relevant training, and one person (1.4%) had received a one-hour relevant training. Only three out of 70 participants (4.3%) had received in-service educational training about CAN and were satisfied with their training. The rest of the participants (42 of 70, 60%) reported having none to minimal (25 of 70, 35.7%) educational preparation in service about dealing with CAN. Approximately 51 (72.9%) participants noted that their preregistration preparation was inadequate to deal with CAN cases, 18 (25.7%) that it was minimal, and only one (1.4%) noted that the preparation was adequate.

Attitudes toward CAN (three components)

Attitudes toward CAN were measured by using three subscales: child discipline, punishment and culpability of the perpetrator, and professional responsibility in child protection. Participants disagreed or strongly disagreed with all attitudes regarding harsh child discipline. The mean score was 34.34 (±2.97) points; since the highest score is 36, the result reflects the strong opposition of midwives to corporal punishment.

Regarding the subscale "punishment and culpability of the perpetrator," the mean score was 25.10 (±4.25) points; since the highest score can be 30, the result reflects the opposition to abusive parents. However, 10 of 70 (14.3%) stated that isolated CAN cases should not be reported to the authorities.

Finally, regarding the subscale "professional responsibility in child protection," almost all midwives (69 of 70, 98.6%) agreed that they should advocate for abused children but more than half (38 of 70, 55.1%) stated that it is very time-consuming to deal with child abuse cases. The mean score for this subscale was 32.60 (±4.91) points; since the highest score can be 42, the result reflects the fair agreement of the participants with their role in reporting.

Knowledge of child abuse and the reporting law

The mean score of knowledge of CAN and the reporting law was 6.63 (±2.32) points, which, given that the highest score can be 13, reflects moderate knowledge. In other words, participants answered correctly to only 51% of the questions. Of note, more than half of midwives were unaware of the Greek legislation about CAN, since 39 of 70 (55.7%) did not know if they were mandatory reporters of CAN, 46 (65.7%) did not know if they could be fined in case they did not report suspected CAN, and 41 (58.6%) stated that they did not know if they could be sued in case they reported suspected CAN, which was not substantiated in court. Additionally, 36 midwives (51.4%) mistakenly believed that most children's sexual abuse involves physical force.

Subjective norms

Forty-four of 70 participants (77%) stated that most people important to them feel that they should report suspected CAN. Accordingly, 61 midwives (87.1%) responded that most people whose opinion they respect consider that they should report suspected CAN. The mean score was 8.69 (±1.61) points, which, since the highest score can be 10, reflects the participants' strong agreement with the statements of the subscale.

Perceived behavioral control

Participants' responses were almost evenly divided regarding the control they feel they have over reporting suspected CAN. Specifically, 25 of 70 (35.7%) felt they did not have any control over reporting, 24 (34.3%) were neutral, and 21 (30.0%) stated they had great control over reporting CAN. Forty-two midwives (60%) felt that they could do something about CAN. However, 36 participants (51.4%) noted that they did not know how to report child abuse, 35 (50.7%) did not have many resources available to report CAN, and 52 (74.3%) agreed with the statement that their professional training did not meet the clinical needs for dealing with CAN. The mean score was 24.37 (±5.10) points, which, since the highest score can be 40, suggests a moderate attitude toward the items that comprise the perceived control subscale behavior.

Intended reporting behavior

The mean score of intended reporting behavior was 32.37 for severe cases and 28.62 for less severe cases, which, since the score can be from 4 to 40, expresses the midwives' tendency to report incidents they consider more severe versus those they consider less severe. The highest score was reached in the severe sexual abuse scenario (9.71 ± 0,89), and the second highest in the severe physical abuse scenario (9.70 ± 0.75) (Table 2).

**Table 2:** Descriptive statistics of intended reporting behavior SD: standard deviation

Intended reporting behavior	Mean	SD	Range
Left child home alone until midnight and child started a fire (severe neglect)	6.66	2.35	1-10
Delay in medical treatment for their child (less severe neglect)	6.61	2.69	1-10
Engage in sexual intercourse with their child (severe sexual abuse)	9.71	0.89	1-10
Dress a girl like a boy and tell her they wanted a boy (severe psychological abuse)	6.30	2.65	1-10
Hit a child’s palms and legs with a cane (less severe physical abuse)	8.37	1.95	1-10
Beat a child resulting in facial bruising and rib fractures (severe physical abuse)	9.70	0.75	1-10
Ridicule and criticize the child (less severe psychological abuse)	5.36	2.46	1-10
Show pornographic pictures (less severe sexual abuse)	8.29	2.16	1-10

Internal consistency, reliability, and validity of CARIS

To test the internal consistency of the six subscales of the CARIS scale, Cronbach's coefficient alpha was calculated, both for the first and the second completion of the questionnaire. Pearson's r coefficient was used to test repeatability, while the ICC and t-test for dependent samples were used to assess test-retest reliability. Table 3 presents the values of the above coefficients and tests, and shows that our results are consistent, stable, and highly repeatable.

**Table 3:** Internal consistency, reliability, and validity of Greek version of CARIS ICC: intraclass correlation coefficient; CI: confidence interval; CARIS: Child Abuse Report Intention Scale ^a^First completion ^b^Second completion

Subscale	Items	Attribute	Measure	Value	Significance
Attitudes toward childbearing belief and discipline	6	Internal consistency	Cronbach's alpha	0.794^a^	-
				0.805^b^	
		Repeatability	Pearson's r	r = 0.797	<0.001
		Test-retest reliability I	ICC (95% CI)	0.794 (0.708-0.862)	<0.001
		Test-retest reliability II	Paired samples t-test	34.34 (±2.97)^a^	0.160
				23.99 (±3.36)^b^	
Attitudes regarding punishment and culpability of offenders or victims of child abuse	5	Internal consistency	Cronbach's alpha	0.640^a^	-
				0.767^b^	
		Repeatability	Pearson's r	r = 0.756	<0.001
		Test-retest reliability I	ICC (95% CI)	0.640 (0.486-0.759)	<0.001
		Test-retest reliability II	Paired samples t-test	24.10 (±4.25)^a^	0.498
				24.84 (±4.76)^b^	
Attitudes regarding professional responsibility	7	Internal consistency	Cronbach's alpha	0.549^a^	-
				0.535^b^	
		Repeatability	Pearson's r	r = 0.777	<0.001
		Test-retest reliability I	ICC (95% CI)	0.549 (0.363-0.697)	<0.001
		Test-retest reliability II	Paired samples t-test	32.50 (±4.88)^a^	0.020
				31.54 (±4.85)^b^	
Knowledge of child abuse and reporting low	13	Internal consistency	Cronbach's alpha	0.700^a^	-
				0.713^b^	
		Repeatability	Pearson's r	r = 0.649	<0.001
		Test-retest reliability I	ICC (95% CI)	0.700 (0.585-0.794)	<0.001
		Test-retest reliability II	Paired samples t-test	6.63 (±2.32)^a^	0.124
				6.27 (±2.27)^b^	
Subjective norms	2	Internal consistency	Cronbach's alpha	0.810^a^	-
				0.842^b^	
		Repeatability	Pearson's r	r = 0.657	<0.001
		Test-retest reliability I	ICC (95% CI)	0.810 (0.695-0.882)	<0.001
		Test-retest reliability II	Paired samples t-test	8.69 (±1.61)^a^	0.858
				8.71 (±1.59)^b^	
Perceived behavioral control over reporting suspicions of child abuse	8	Internal consistency	Cronbach's alpha	0.694^a^	-
				0.686^b^	
		Repeatability	Pearson's r	r = 0.726	<0.001
		Test-retest reliability I	ICC (95% CI)	0.694 (0.570-0.793)	<0.001
		Test-retest reliability II	Paired samples t-test	24.37 (±5.13)^a^	0.770
				24.24 (±4.98)^b^	

Subsequently, internal consistency, validity, and reliability of the subscale "Intended Practice Behaviors: Vignettes questions and Respond" of CARIS were assessed. Table 4 shows that the results are consistent, stable, and highly repeatable.

**Table 4:** Internal consistency, reliability, and validity of the subscale “intended practice behaviors: vignettes questions and respond options” ICC: intraclass correlation coefficient; CI: confidence interval; S: severe; LS: less severe ^a^First completion ^b^Second completion

Subscales	Attribute	Measure	Value	Significance
Intended reporting behavior (S)	Internal consistency	Cronbach's alpha	0.527^a^	-
			0.716^b^	
	Repeatability	Pearson's r	0.850	<0.001
	Test-retest reliability I	ICC (95% CI)	0.527 (0.317-0.685)	<0.001
	Test-retest reliability II	Paired samples t-test	32.37 (±4.76)^a^	0.176
			32.84 (±5.47)^b^	
Intended reporting behavior (LS)	Internal consistency	Cronbach's alpha	0.748^a^	-
			0.755^b^	
	Repeatability	Pearson's r	0.831	<0.001
	Test-retest reliability I	ICC (95% CI)	0.748 (0.635-0.832)	<0.001
	Test-retest reliability II	Paired samples t-test	28.63 (±7.03)^a^	0.022
			29.77 (±6.98)^b^	
Knowledge (S)	Internal consistency	Cronbach's alpha	0.683^a^	-
			0.715^b^	
	Repeatability	Pearson's r	0.878	<0.001
	Test-retest reliability I	ICC (95% CI)	0.683 (0.557-0.784)	<0.001
	Test-retest reliability II	Paired samples t-test	69.8 (±7.15)^a^	0.061
			70.6 (±7.03)^b^	
Knowledge (LS)	Internal consistency	Cronbach's alpha	0.737^a^	-
			0.723^b^	
	Repeatability	Pearson's r	0.841	<0.001
	Test-retest reliability I	ICC (95% CI)	0.737 (0.632-0.821)	<0.001
	Test-retest reliability II	Paired samples t-test	64.6 (±9.22)^a^	0.002
			66.5 (±8.35)^b^	
Attitude (S)	Internal consistency	Cronbach's alpha	0.863^a^	-
			0.887^b^	
	Repeatability	Pearson's r	0.822	<0.001
	Test-retest reliability I	ICC (95% CI)	0.863 (0.808-0.907)	<0.001
	Test-retest reliability II	Paired samples t-test	53.2 (±16.41)^a^	0.429
			51.2 (±16.57)^b^	
Attitude (LS)	Internal consistency	Cronbach's alpha	0.890^a^	-
			0.877^b^	
	Repeatability	Pearson's r	0.803	<0.001
	Test-retest reliability I	ICC (95% CI)	0.890 (0.846-0.925)	<0.001
	Test-retest reliability II	Paired samples t-test	51.4 (±17.02)^a^	0.216
			53.0 (±16.14)^b^	

Relationship between attitudes, knowledge, subjective norm, perceived behavioral control, and intention to report. Further analysis showed that attitudes (Spearman's rho = 0.555; p < 0.001), subjective norm (Spearman's rho = 0.228; p < 0.015), and perceived behavioral control (Spearman's rho = 0.494; p <0.001) are moderately positively correlated with the midwives' intention to report suspected CAN. In contrast, no correlation emerged between the participants' knowledge and their intention to report (Spearman's rho = -0.082; p = 0.501).

## Discussion

While midwives' contribution to child protection is a common assumption, little is known about the factors influencing their intention to report CAN. The main purpose of this explorative study was to translate and validate a Greek version of the CARIS instrument. The outcomes confirmed that the Greek CARIS can be used by midwives since the psychometric results showed consistency, stability, and high repeatability of the tool. Compared to the original CARIS [9,16], some differences were identified, perhaps due to the cultural and educational dissimilarities, such as the higher knowledge score reached by Taiwanese nurses compared to Greek midwives and the almost zero tolerance to harsh punishment as a means of child discipline shown by Greek midwives compared to Taiwanese nurses.

A key finding was the lack of training for Greek midwives in dealing with CAN cases. In more detail, the great majority (66 of 70, 94.3%) received zero training about CAN in midwifery school, while 51 (73%) participants noted that their general educational preparation was inadequate to deal with CAN cases. Most of the participants (67 of 70, 95.7%) reported that their in-service training was zero to minimal. Our results agree with findings from previous studies in Taiwan and Saudi Arabia [9,11]. Characteristically, Lee et al. [20] pointed out that 84.9% of Taiwanese nurses had never taken any CAN course during their undergraduate studies, and 80.7% had never received in-service training, proving the universal need to include training about early identification and reporting of suspected CAN cases with emphasis on the reporting law for health care providers worldwide.

Another interesting finding that emerged from our study is the significant difference between the actual reporting and the intention to report. Only seven of 70 midwives (10%) had reported suspected CAN cases, while the mean score of intention to report severe cases of CAN was 32.3/40 and 28.6/40 for less severe cases. Moreover, 12 participants (17%) stated that although they had suspected CAN in the past, they did not report it. The low percentage of reporting is consistent with the results of Salami and Alhalal [11], where only 6.6% of the correspondents had ever reported a suspected CAN case, while another 5.5% suspected a CAN case but never reported it. Similarly, 70% of Taiwanese nurses had never reported a suspected CAN case [20], and only 14% of them had reported one or more cases of CAN in the past [9].

In our study, the main reasons for underreporting were the feeling of uncertainty about the plausibility of the evidence and the lack of trust in the legal authorities. Similar responses are commonly found in the literature [9-11,16,18-19], demonstrating that it is important to have joint training programs for health professionals and other professionals directly responsible for managing CAN cases, such as police or law enforcement personnel. In this way, health professionals' trust in legal authorities can be improved. In Greece, in 2024, there was a strengthening of the legislation regarding the mandatory reporting of suspected cases of CAN by professionals such as doctors or teachers. Moreover, according to the new law, these professionals will have immunity in case of unsubstantiated reports. Unfortunately, midwives are not included in this law.

Regarding attitudes toward CAN, our respondents had strong negative attitudes toward corporal punishment and toward parents who act abusively to their children, and positive attitudes toward professional liability of reporting suspected CAN. These results align with other studies in East Asia despite the traditional Chinese culture that accepts corporal punishment to instill filial piety [16,19]. On the other hand, Salami and Alhalal [11] found that 89.5% of nurses in Saudi Arabia agreed with the statement that "corporal punishment is an effective childbearing practice." Their interpretation was that Arab cultures tend to use corporal punishment as a means of disciplining children [22,27]. Today, in Greece, the use of harsh discipline methods against children is impermissible, reprehensible, and illegal. It was in 2006 that a legislative regulation was adopted to protect children from domestic violence by criminalizing any corporal punishment whatsoever against children (law 3005/2006). In a survey conducted by Papadakou et al., 27.36% of Greek parents in 2014 continued to use physical punishment against their children, compared to 2001, when that percentage was 82.85% [28]. This finding suggests that it takes time for generations to change their parenting tactics, but it is possible. As midwives, we need to foster positive parenting and bonding between parents and their children, starting early in the pregnancy period, when our role is crucial.

Our results are discouraging regarding midwives' knowledge of CAN and the reporting law. Respondents answered only 51% of the questions correctly, leading to a mean score of 6.63 out of 13. Unfortunately, similar results have been reported worldwide. Similar scores were found for nurses in Hong Kong [18]. Nurses in Saudi Arabia scored even lower with a mean score of 5.2/13 [11], Korean nursing students had a mean score of 7/13 [21], Israeli nurses and doctors also had a low score regarding their level of knowledge [22], and nurses from the Philippines answered correctly in fewer than 50% of the relative to CAN and the reporting law questions [19]. In contrast, Taiwanese nurses had a higher score of knowledge, answering 60% of the relevant questions correctly, while Australian nurses were confident and knowledgeable about CAN and the reporting laws, answering 72%-90% correctly [10]. In both Taiwan and Australia, nurses are authorized reporters of suspected CAN cases, while in Greece, midwives are not legally required to report suspected CAN cases. This may be one of the reasons for the large difference in the level of knowledge of CAN and the reference law between Greek midwives and Taiwanese and Australian nurses. As mentioned earlier, midwives are unfortunately not included in the recent law regarding the mandatory reporting of suspected cases of CAN and the immunity of professionals in case of unsubstantiated reports. This is a legal gap that needs to be addressed.

Regarding midwives' intention to report suspected CAN, we found that they were more likely to report severe cases than less severe cases of CAN (scores of 32.37 out of 40 and 28.62 out of 40, respectively). In fact, the severe sexual abuse cases reached the highest reporting score, while the severe physical abuse scenario was the second highest. However, our respondents were less likely to report psychological abuse cases regardless of severity. These results are consistent with similar findings in other studies [10,11,16,18,19]. Further analyzing participants' responses to offered scenarios, midwives were moderately to fairly likely to report the incident about the sick, dehydrated 10-month-old infant (6.61 ± 2.69) (second scenario, Table 2). This was an unexpected finding since this scenario is close to midwifery's everyday practice in Greece, and it was presumed that midwives would be more sensitive to this sort of CAN. This finding indicates the need to adequately train midwives to recognize, report, and deal with CAN cases.

Finally, the most influential predictor of intention to report suspected CAN was the "attitude toward child abuse," while no significant correlation emerged between "knowledge" and intention to report. "Attitude" was the strongest positive relation to "intention to report" in several previous studies as well [19,22,29,30], while no correlation between "knowledge" and reporting behavior was mentioned in other studies too [18,19,21,22]. In contrast, knowledge was the most significant predictive variable for intention to report in the studies by Feng and Levine [16] and Salami and Alhalah [11].

Limitations and future directions

This study is the first to validate the CARIS tool among Greek midwives, contributing essential data to support CAN reporting interventions. Our study, however, bears some limitations, starting with the small, convenience sample, which may not be representative of the population of Greek midwives. The area of our target population is, however, the capital of Greece, where the great majority of midwives are employed since it also has the largest population of residents. A larger and more representative sample, including different areas in Greece, would have strengthened our study. But, as mentioned earlier, this is a pilot study that proceeds with a larger one. In addition, the questionnaire was self-reported, and due to the sensitivity of its content and social desirability, some of the responses may be biased. Future studies should aim for larger, representative samples across Greece and consider longitudinal designs to further explore the predictors of CAN reporting behavior. Further research is needed on whether interventions targeting these predictors produce actual behavior change. As previously mentioned, Greek midwives are not mandated reporters of suspected CAN cases, which may affect their intention to report such cases. Beyond the imperative need for midwives to be legally obliged to report suspected cases of CAN, future studies can be designed to test the effect this implementation may have on midwives' intention to report respective cases.

## Conclusions

Child abuse is a widespread problem with undeniable short- and long-term consequences. The role of midwives in preventing, identifying, and reporting CAN is primary and critical to children's well-being. Our study demonstrated that the Greek version of CARIS is reliable and a valid tool to be used to determine the factors that influence midwives in reporting cases of suspected CAN.

This is the first time that factors influencing midwives' attitudes toward reporting CAN have been studied in Greece. This study found significant associations between midwives' intention to report CAN and factors such as attitudes, perceived behavioral control, and subjective norms. More specifically, we found that Greek midwives have strong negative attitudes toward harsh child discipline, low tolerance for perpetrators, positive attitudes toward the responsibility to report suspected CAN, and moderate knowledge of CAN, and are more likely to report severe CAN cases than less severe ones. In general terms, similar results were found for nurses in East Asia, and Australian and Taiwanese nurses had higher knowledge of CAN, maybe due to their mandatory role as CAN reporters in their countries. Given the cross-sectional design, further research is needed to explore causative links and assess intervention effectiveness.

Finally, one of our key findings was the lack of adequate and appropriate training in dealing with CAN cases, both at undergraduate and postgraduate levels. This finding was paramount in other similar studies worldwide. Health policy makers and midwifery program educators need to address these issues and provide the necessary training programs for midwives to enable them to play their critical role as advocates for women and children.
